# Characterization of Antibiotic Resistance Profiles in the Oral Microbiota of the Pakistani Population

**DOI:** 10.7759/cureus.72617

**Published:** 2024-10-29

**Authors:** Javeria Zaheer, Muhammad Naeem Khan, Atiq Ur Rahman, Muhammad Ishfaq, Muhammad Asif Shahzad, Madeeha Lateef, Sudhair Abbas Bangash

**Affiliations:** 1 Department of Microbiology, Akhtar Saeed Medical and Dental College, Lahore, PAK; 2 Department of Ear, Nose, and Throat (ENT), Gomal Medical College, Dera Ismail Khan, PAK; 3 Department of Maxillofacial Surgery, Gomal Medical College, Dera Ismail Khan, PAK; 4 Department of Oral and Maxillofacial Surgery, College of Dentistry, King Khalid University, Abha, SAU; 5 Department of Oral and Maxillofacial Surgery, Multan Medical and Dental College, Multan, PAK; 6 Department of Oral and Maxillofacial Surgery, Azra Naheed Dental College, The Superior University, Lahore, PAK; 7 Department of Biochemistry, Sardar Begum Dental College, Gandhara University, Peshawar, PAK; 8 Department of Pharmacy, Sarhad University of Science and Information Technology, Peshawar, PAK

**Keywords:** antibiotic resistance, erythromycin, oral microbiota, pakistan, penicillin, staphylococcus aureus, streptococcus mutans, tetracycline

## Abstract

Background

The oral microbiota's resistance to antibiotics presents a serious threat to world health, especially in developing nations where misuse of antibiotics is common.

Objective

The objective of this study was to characterize the antibiotic resistance profiles in the oral microbiota of Pakistani adults.

Methodology

The Department of Microbiology at Akhtar Saeed Medical and Dental College in Lahore, Pakistan, carried out a cross-sectional study from January 2022 to December 2022. Oral swabs were collected from 240 adults (aged 18 and older) who had not used antibiotics in the past three months. The disk diffusion method was used for both antibiotic susceptibility testing and bacterial cultures. Descriptive statistics and logistic regression analysis were conducted using IBM SPSS Statistics for Windows, Version 25 (Released 2017; IBM Corp., Armonk, New York, United States) to examine associations within the demographic data.

Results

The study examined 240 participants, comprising 133 students (55.42%), 64 professionals (26.67%), and 43 individuals in other occupations (17.92%). Of the participants, 128 were male (53.33%) and 112 were female (46.67%). With 81 isolates (33.75%), *Streptococcus mutans* was the most common species of bacterium, followed by *Staphylococcus aureus *with 69 isolates (28.75%). The majority of cases (n = 72; 30.00%) were resistant to penicillin, followed by erythromycin (22.50%) in 54 instances and tetracycline (19.58%) in 47 cases. Age group (50 years and above; β = 0.120, OR = 1.128, p = 0.017), penicillin resistance (β = 0.150, OR = 1.162, p = 0.001), erythromycin resistance (β = 0.120, OR = 1.128, p = 0.013), and ciprofloxacin resistance (β = 0.130, OR = 1.139, p = 0.014) were all significantly associated with the results of the regression analysis. Additionally, resistance was positively associated with the occupation "student" (β = 0.110, OR = 1.116, p = 0.047).

Conclusion

The high levels of antibiotic resistance observed, particularly in older age groups and certain occupations, underscore the urgent need for enhanced antibiotic stewardship and regulatory measures in Pakistan.

## Introduction

A serious worldwide health issue that is making treating infectious illnesses more difficult is antibiotic resistance [1,2]. The emergence of resistant bacterial strains reduces the efficacy of currently available medicines, leading to longer disease durations, higher death rates, and rising medical expenses [3-5]. Oral and systemic health depend on the oral microbiota, which is made up of many microbial communities that live in the mouth cavity [6]. Antibiotic-resistant bacteria have emerged in these areas nonetheless, as a result of the widespread and sometimes careless use of antibiotics [7]. In underdeveloped nations like Pakistan, where antibiotic abuse is common and regulatory control is lax, this problem is especially concerning [8].

The easy access to over-the-counter antibiotics and the lack of strict criteria for prescribing them both contribute to the abuse and overuse of antibiotics in Pakistan [9]. As a result, there is strong selection pressure on the oral microbiota in Pakistani populations, which promotes the growth of resistant species [10]. Developing focused methods to stop the development of resistance and improve antibiotic stewardship requires an understanding of the resistance profiles of the oral microbiota [11].

Prior studies have mostly focused on clinical isolates of antibiotic-resistant infections, with very little attention paid to the commensal microbiota of the oral cavity [12]. However, the oral microbiota may serve as a source of resistance genes that can spread harmful bacteria and exacerbate the resistance issue [13]. Data on the antibiotic resistance profiles of oral microbiota in Pakistani populations are conspicuously lacking, despite the crucial relevance of this problem. This knowledge gap highlights the critical necessity for further investigations to clarify the resistance patterns in this population's oral microbiome.

Research objective

The objective of this study was to characterize the antibiotic resistance profiles in the oral microbiota of Pakistani adults, with a focus on identifying key demographic factors that may influence resistance patterns.

## Materials and methods

Study design and settings

This cross-sectional study was conducted at the Department of Microbiology, Akhtar Saeed Medical and Dental College, Lahore, Pakistan, over a one-year period from January 2022 to December 2022. The study utilized a stratified random sampling technique to ensure representation across key demographic characteristics, including age, gender, and occupation, thus reducing selection bias. Participants aged 18 years and older who had not used antibiotics in the previous three months were included to minimize the impact of recent antibiotic usage on the oral microbiota. This design facilitated a comprehensive analysis of antibiotic resistance patterns, allowing for a thorough examination of how demographic factors influence resistance profiles. By addressing potential biases in participant selection, this study aimed to produce reliable and generalizable findings relevant to public health initiatives.

Inclusion and exclusion criteria

The study included participants aged 18 years and older who had not used antibiotics in the previous three months to minimize the potential influence of recent antibiotic use on the oral microbiota. Exclusion criteria were designed to ensure a homogeneous sample and included individuals on immunosuppressive treatment, those with systemic disorders, and women who were pregnant or nursing.

Sample size and sampling technique

The research included a total of 240 participants, selected using a stratified random sampling technique to ensure representation across key demographic variables such as age, gender, and occupation. The population was categorized into strata based on these variables, and participants were randomly selected from each stratum to obtain a sample reflective of the broader community. This approach minimized selection bias and enhanced the validity of the findings, allowing for more accurate analysis of antibiotic resistance patterns within the oral microbiota.

Data collection

Data were systematically collected using oral swabs from each of the 240 adult participants. All samples were obtained using sterile, single-use swabs to maintain aseptic conditions and prevent cross-contamination. To optimize sample quality, participants were instructed to abstain from eating, drinking, or performing oral hygiene practices for at least one hour before sample collection. A trained healthcare professional collected the swabs by gently sampling the inside of each participant's cheeks, gums, and tongue areas known to be primary reservoirs of oral microbiota.

The swabs were promptly transferred into sterile transport media tubes immediately after collection to preserve sample integrity during transit to the microbiology lab. The samples were then processed in line with Clinical and Laboratory Standards Institute (CLSI) guidelines. Selective media plates were used to culture the bacteria commonly found in the oral cavity, followed by incubation. The bacterial identification was carried out using standard biochemical tests.

Antibiotic susceptibility testing

Antibiotic susceptibility testing employed the Kirby-Bauer disk diffusion method. Bacterial isolates were exposed to disks impregnated with antibiotics, including penicillin, erythromycin, tetracycline, ciprofloxacin, and vancomycin, following CLSI guidelines. Zones of inhibition were measured post-incubation to assess resistance profiles.

Demographic and lifestyle data collection

Demographic data, including age, gender, and occupation, as well as lifestyle factors such as smoking status, oral hygiene practices, and frequency of dental visits, were systematically recorded at the time of sample collection. Participants were categorized into age groups: 18-29, 30-39, 40-49, and 50 years and above. Gender distribution was recorded as either male or female. For occupational classification, participants were identified as students, professionals, or from other categories, including skilled and unskilled workers, homemakers, and retired individuals.

Smoking status was documented, distinguishing between smokers and non-smokers. Participants were also asked about their oral hygiene practices, with specific attention given to the frequency of brushing, which categorized individuals into those with regular oral hygiene practices and those with irregular or no brushing habits. This comprehensive collection of demographic and lifestyle data aimed to facilitate a better understanding of the potential influences these factors may have on antibiotic resistance patterns within the oral microbiota.

Laboratory findings and resistance profiles

Bacterial species were isolated from the oral swabs collected from participants using selective media plates. Following collection, samples were processed according to CLSI guidelines to ensure accurate identification and characterization of the oral microbiota. The isolated bacterial species were subjected to a series of standard biochemical tests to determine their identity.

Antibiotic susceptibility testing was conducted using the Kirby-Bauer disk diffusion method, where bacterial isolates were exposed to disks impregnated with various antibiotics. This methodology involved measuring the zones of inhibition around the antibiotic disks after incubation to assess the resistance profiles of the isolated bacteria. This process allowed for a comprehensive evaluation of antibiotic resistance patterns within the sampled population, focusing on common antibiotics used in clinical practice.

Demographic factors, such as age, gender, and occupation, were taken into account during the analysis of the antibiotic resistance profiles, facilitating a thorough understanding of how these variables influence bacterial resistance in the oral microbiota.

Statistical analysis

Data were analyzed using IBM SPSS Statistics for Windows, Version 25 (Released 2017; IBM Corp., Armonk, New York, United States). Descriptive statistics summarized demographic variables and resistance profiles, while logistic regression analysis assessed associations between antibiotic resistance (penicillin, erythromycin, tetracycline, ciprofloxacin, and vancomycin) and demographic factors (age, gender, and occupation). The results revealed statistically significant associations between resistance and certain demographics' age.

Ethical approval

The study protocol was approved by the Institutional Review Board (IRB) of Akhtar Saeed Medical and Dental College (Reference No.: 03-AMDC/ADM/2021). Written informed consent was obtained from all participants prior to sample collection. This research adhered to the ethical principles outlined in the Declaration of Helsinki.

## Results

A wide range of demographic characteristics were represented among the 240 participants in the research (Table 1). Of the 240 participants, 84 (35.00%) were in the 18-29 age range, 66 (27.50%) were in the 30-39 age range, 49 (20.42%) were in the 40-49 age range, and 41 (17.08%) were in the 50+ age range. The gender distribution was relatively balanced, with 128 males (53.33%) and 112 females (46.67%). Regarding occupation, 133 participants (55.42%) were students, 64 (26.67%) were professionals, and 43 (17.92%) were engaged in other occupations. Additionally, 52 participants (21.67%) reported being smokers, and 188 (78.33%) were non-smokers. Most participants (83.33%) practiced regular oral hygiene, while 16.67% reported irregular or no brushing habits.

**Table 1:** Demographic characteristics of participants (n = 240) *The other occupation category includes skilled workers (n = 18, 7.50%), unskilled workers (n = 10, 4.17%), homemakers (n = 9, 3.75%), and retired individuals (n = 6, 2.50%).

Demographic variable	Category	Number of participants (n)	Percentage (%)
Age	18-29 years	84	35
	30-39 years	66	27.5
	40-49 years	49	20.42
	50 years and above	41	17.08
Gender	Male	128	53.33
	Female	112	46.67
Occupation	Student	133	55.42
	Professional	64	26.67
	Other*	43	17.92
Education level	Primary/secondary	47	19.58
	College	123	51.25
	Graduate/postgraduate	70	29.17
Smoking status	Smoker	52	21.67
	Non-smoker	188	78.33
Oral hygiene practices	Regular brushing	200	83.33
	Irregular/no brushing	40	16.67
Dental visits	Regular	115	47.92
	Occasional/never	125	52.08

**Figure 1:**
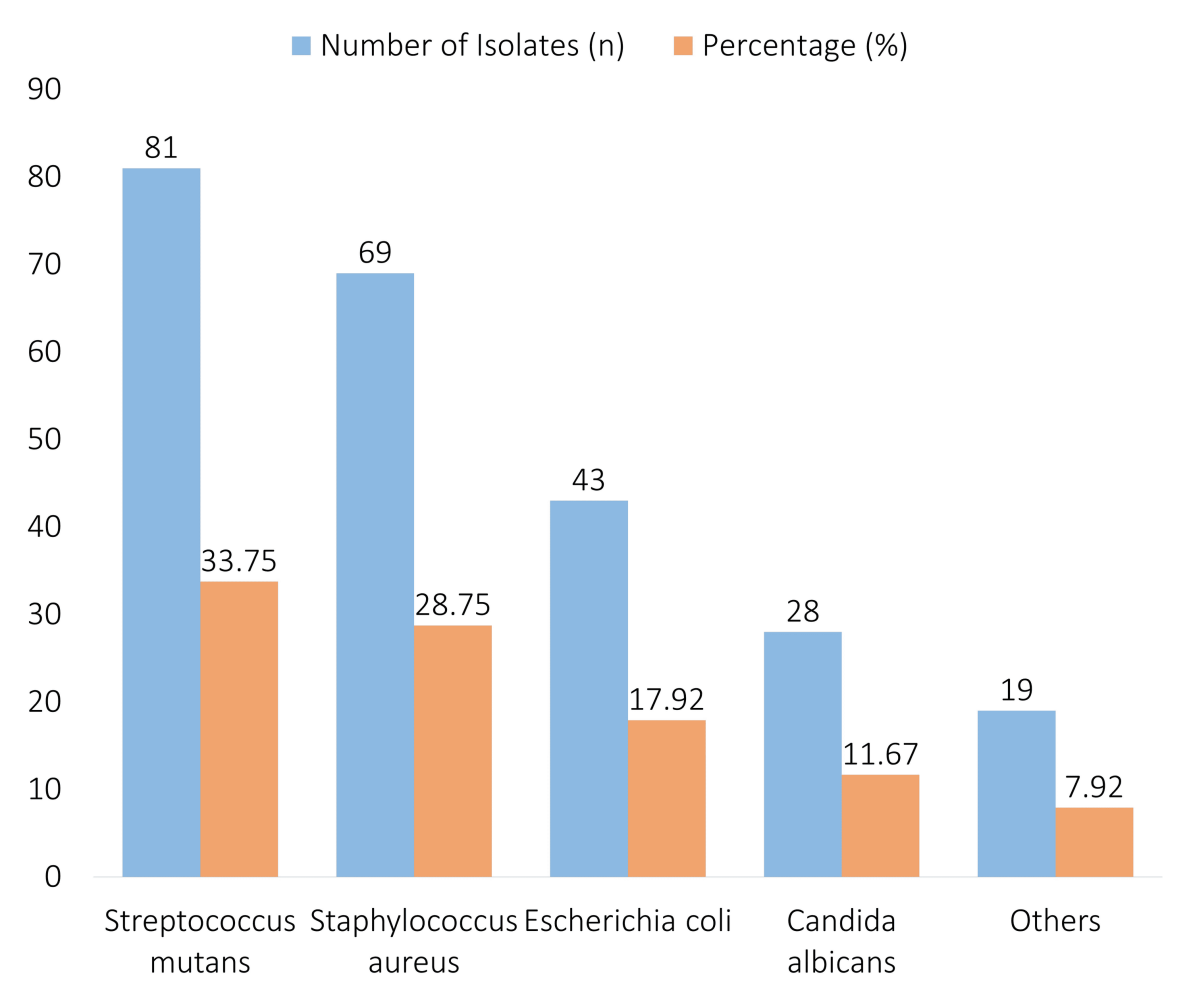
Distribution of bacterial species detected

According to the distribution of bacterial species found in the isolates, *Streptococcus mutans* was the most common, accounting for 81 isolates (33.75%). In second place, with 69 isolates (28.75%), was *Staphylococcus aureus*. *Candida albicans* was found in 28 isolates (11.67%), while *Escherichia coli* was found in 43 isolates (17.92%). It was determined that 19 isolates (7.92%) belonged to a different species (Figure 1).

**Figure 2:**
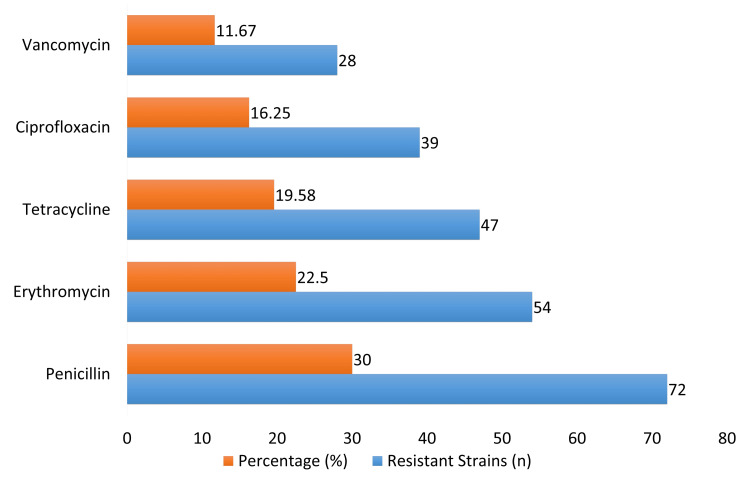
Antibiotic resistance patterns

Penicillin was found to be the most prevalent resistant strain, accounting for 72 instances (30.00%), according to the study of antibiotic resistance patterns in Figure 2. Tetracycline was found in 47 instances (19.58%), while erythromycin followed in 54 cases (22.50%). There were 39 instances (16.25%) with ciprofloxacin resistance and 28 cases (11.67%) with vancomycin resistance.

Antibiotic resistance profiles are shown by age group in Table 2. There were 84 individuals in the 18-29 age range, and the resistance rates for penicillin, erythromycin, tetracycline, ciprofloxacin, and vancomycin were 24 (28.57%), 20 (23.81%), 18 (21.43%), 16 (19.05%), and 6 (7.14%), respectively. The group of 66 individuals aged 30-39 showed resistance in the following ways: penicillin was given to 18 (27.27%), erythromycin to 14 (21.21%), tetracycline to 12 (18.18%), ciprofloxacin to 11 (16.67%), and vancomycin to 11 (16.67%). The resistance rates were as follows: 15 (30.61%) to penicillin, 10 (20.41%) to erythromycin, 10 (20.41%) to tetracycline, 8 (16.33%) to ciprofloxacin, and 6 (12.24%) to vancomycin in the group of 49 individuals aged 40 to 49. Of the 41 individuals who were 50 years of age or older, 15 (36.59%) had resistance to penicillin, 10 (24.39%) to erythromycin, 7 (17.07%) to tetracycline, 4 (9.76%) to ciprofloxacin, and 5 (12.20%) to vancomycin at baseline.

**Table 2:** Resistance profiles by age group

Age group	Penicillin	Erythromycin	Tetracycline	Ciprofloxacin	Vancomycin
18-29 years (n; %)	24 (28.57)	20 (23.81)	18 (21.43)	16 (19.05)	6 (7.14)
30-39 years (n; %)	18 (27.27)	14 (21.21)	12 (18.18)	11 (16.67)	11 (16.67)
40-49 years (n; %)	15 (30.61)	10 (20.41)	10 (20.41)	8 (16.33)	6 (12.24)
50 years and above (n; %)	15 (36.59)	10 (24.39)	7 (17.07)	4 (9.76)	5 (12.20)

The patterns of antibiotic resistance broken down by gender are shown in Table 3. Thirty-three (25.78%) of the 128 male participants were resistant to erythromycin, 30 (23.44%) to tetracycline, 27 (21.09%) to ciprofloxacin, and 16 (12.50%) to penicillin. The 112 female participants showed resistance to penicillin in 39 cases (34.82%), erythromycin in 24 cases (21.43%), tetracycline in 20 cases (17.86%), ciprofloxacin in 17 cases (15.18%), and vancomycin in 12 cases (10.71%).

**Table 3:** Resistance profiles by gender

Gender	Penicillin	Erythromycin	Tetracycline	Ciprofloxacin	Vancomycin
Male (n; %)	33 (25.78)	30 (23.44)	27 (21.09)	22 (17.19)	16 (12.50)
Female (n; %)	39 (34.82)	24 (21.43)	20 (17.86)	17 (15.18)	12 (10.71)

The profiles of antibiotic resistance by occupation are shown in Table 4. About 133 children tested positive for 39 (29.32%) penicillin resistance, 33 (24.81%) erythromycin resistance, 25 (18.80%) tetracycline resistance, 22 (16.54%) ciprofloxacin resistance, and 14 (10.53%) vancomycin resistance. About 64 professionals were found to be resistant to penicillin (19 (29.69%)), erythromycin (13 (20.31%)), tetracycline (12 (18.75%)), ciprofloxacin (12 (18.75%)), and vancomycin (8 (12.50%)). Of the 43 people in the other occupational groups, 8 (18.60%), 10 (23.26%), and 5 (11.63%) were resistant to erythromycin, penicillin, ciprofloxacin, and vancomycin, respectively.

**Table 4:** Association between resistance and occupation

Occupation	Penicillin	Erythromycin	Tetracycline	Ciprofloxacin	Vancomycin
Student (n; %)	39 (29.32)	33 (24.81)	25 (18.80)	22 (16.54)	14 (10.53)
Professional (n; %)	19 (29.69)	13 (20.31)	12 (18.75)	12 (18.75)	8 (12.50)
Other (n; %)	14 (32.56)	8 (18.60)	10 (23.26)	5 (11.63)	6 (13.95)

The variable "age group (50 years and above)" shows a strong positive association with antibiotic resistance in the regression analysis of antibiotic resistance profiles, with a coefficient of β = 0.120 (standard error = 0.05, t-value = 2.4, p = 0.017) and an odds ratio (OR) of 1.128. This indicates that individuals aged 50 years and above exhibit higher levels of antibiotic resistance compared to other age groups. Significant associations with increased resistance are also observed in "penicillin resistance" (β = 0.150, standard error = 0.045, OR = 1.162, t-value = 3.33, p = 0.001), "erythromycin resistance" (β = 0.120, standard error = 0.048, OR = 1.128, t-value = 2.5, p = 0.013), and "ciprofloxacin resistance" (β = 0.130, standard error = 0.052, OR = 1.139, t-value = 2.5, p = 0.014). Resistance associated with the occupation "student" is also positively related to resistance (β = 0.110, standard error = 0.055, OR = 1.116, t-value = 2, p = 0.047). However, resistance to vancomycin does not show a significant association (β = -0.060, standard error = 0.055, OR = 0.942, t-value = -1.09, p = 0.278). Additionally, "tetracycline resistance" demonstrates a moderate positive association (β = 0.100, standard error = 0.05, OR = 1.105, t-value = 2, p = 0.05, Table 5).

**Table 5:** Logistic regression analysis of antibiotic resistance profiles

Variable	Coefficient (β)	Standard error	Odds ratio (OR)	t-value	p-value
Age group (50 years and above)	0.12	0.05	1.128	2.4	0.017
Gender (female)	-0.08	0.06	0.923	-1.33	0.185
Occupation (student)	0.11	0.055	1.116	2	0.047
Penicillin resistance	0.15	0.045	1.162	3.33	0.001
Erythromycin resistance	0.12	0.048	1.128	2.5	0.013
Ciprofloxacin resistance	0.13	0.052	1.139	2.5	0.014
Tetracycline resistance	0.1	0.05	1.105	2	0.05
Vancomycin resistance	-0.06	0.055	0.942	-1.09	0.278

## Discussion

The goal of the research was to describe the profiles of antibiotic resistance in Pakistan's oral microbiota. The results provided important new information on resistance trends and demographic factors. The distribution of bacterial species and resistance profiles among various age groups, genders, and professions was shown by the data. The most common species (33.75%) was *S. mutans*, which is in line with other studies that have shown this species to be predominant in the oral microbiota [14]. Following (28.75%) was *S. aureus*, which is consistent with results from previous research that show it is often isolated from oral samples [15].

Given that penicillin has historically been effective against a wide variety of bacteria, the significant level of resistance to the antibiotic - observed in 30.00% of isolates - reflects a severe worry. This discovery supports the findings of Bilal et al. [16], who also found that bacterial strains from Pakistan have increased penicillin resistance, most likely as a consequence of widespread antibiotic overuse. Twenty-five percent of the isolates had erythromycin resistance, which is consistent with findings from previous studies looking at oral microbiota in areas with comparable antibiotic stewardship issues [17]. Compared to penicillin and erythromycin, tetracycline resistance was lower (19.58%), but still considerable. According to earlier studies, the overprescription and broad-spectrum usage of tetracycline often lead to the emergence of resistance [18].

In comparison to younger groups, the 50-year-old and above group had a greater prevalence of penicillin resistance (36.59%) according to the age-related resistance profiles. This pattern is consistent with research by Wang et al. [19], which links prolonged antibiotic use to higher resistance in elderly populations. There were clear gender disparities as well, with 34.82% of females and 25.78% of men exhibiting penicillin resistance. This discrepancy is consistent with research by Pham-Duc and Sriparamananthan [20], who found comparable gender-related variations in antibiotic resistance trends.

Students showed a 29.32% resistance rate to penicillin, indicating the possible influence of lifestyle and healthcare access on resistance profiles. Occupation-related resistance varied. This conclusion is consistent with the earlier research [21], which found a relationship between professional and lifestyle characteristics and patterns of antibiotic resistance in several demographic groups. The results of the regression analysis supported the involvement of the age group (β = 0.150, p = 0.001) and occupation (β = 0.110, p = 0.047) in the development of penicillin resistance by showing a substantial association between the two variables. This finding is consistent with other research showing that these relationships are essential to comprehending resistance processes and creating focused therapies [22].

Overall, the results of this research on antibiotic resistance in oral microbiota highlight the need for better public health initiatives and antibiotic stewardship to successfully combat resistance. The study's significant resistance rates and demographic differences point to important areas for further investigation and action.

Study limitations

The cross-sectional methodology of this research limits the capacity to deduce causation and temporal association between antibiotic resistance and demographic variables. Recall bias may also be introduced when recent antibiotic usage is determined only by self-reported data. The fact that the research was limited to a single medical institution may also have limited the results' applicability to larger groups. To overcome these constraints and strengthen the validity of the results, future studies should take multi-center strategies, bigger sample numbers, and longitudinal designs into account.

## Conclusions

This research underscores the significant issue of antibiotic resistance in the oral microbiota of Pakistani adults, revealing notable resistance to key antibiotics such as erythromycin and penicillin, with varying resistance patterns across age, gender, and occupational groups. The findings emphasize the urgent need for targeted public health interventions, improved antibiotic stewardship, and stricter regulatory measures in Pakistan. Future studies should delve into the underlying mechanisms of resistance within the oral microbiota to enhance prevention strategies and inform broader public health policies.
